# Combination of artesunate and WNT974 induces KRAS protein degradation by upregulating E3 ligase ANACP2 and β-TrCP in the ubiquitin–proteasome pathway

**DOI:** 10.1186/s12964-022-00834-2

**Published:** 2022-03-19

**Authors:** Rui-Hong Gong, Minting Chen, Chunhua Huang, Hoi Leong Xavier Wong, Hiu Yee Kwan, Zhaoxiang Bian

**Affiliations:** Centre for Cancer and Inflammation Research, School of Chinese Medicine, Hong Kong Baptist University, 7 Baptist University Road, Kowloon Tong, Hong Kong, China

**Keywords:** Colorectal cancer, KRAS, Artesunate, WNT974, Combination treatment, Protein degradation

## Abstract

**Background:**

KRAS mutation is one of the dominant gene mutations in colorectal cancer (CRC). Up to present, targeting KRAS for CRC treatment remains a clinical challenge. WNT974 (LGK974) is a porcupine inhibitor that interferes Wnt signaling pathway. Artesunate (ART) is a water-soluble semi-synthetic derivative of artemisinin.

**Methods:**

The synergistic effect of ART and WNT974 combination in reducing CRC cell viability was determined by the 3-(4,5-dimethylthiazol-2-yl)-2,5-diphenyltetrazolium bromide (MTT) assay. RT-PCR was utilized for the mRNA levels of KRAS, CUL7, ANAPC2, UBE2M, RNF123, SYVN1, or β-TrCP. Western blot assay was utilized for the protein levels of NRAS, HRAS, KRAS, ANAPC2, β-TrCP, GSK-3β, p-Akt (Ser473), t-Akt, p-PI3K (Tyr458), t-PI3K, p-mTOR (Ser2448), t-mTOR. Xenograft mouse model assay was performed for the anti-CRC effect of combination of ART and WNT974 in vivo. IHC assay was utilized for the levels of KRAS, β-TrCP, GSK-3β or ANAPC2 in tumor tissues.

**Results:**

Our study shows that the combination of WNT974 and ART exhibits synergistic effect in reducing CRC growth. The combination treatment significantly reduces KRAS protein level and activity in CRC cells. Interestingly, the combination treatment increases E3 ligases ANAPC2 expression. Our data show that overexpression of ANAPC2 significantly reduces KRAS protein levels, which is reversed by MG132. Knockdown of ANAPC2 in CRC abolishes the combination treatment-reduce KRAS expression. Besides, the treatment also increases the expressions of GSK-3β and E3 ligase β-TrCP that is known to degrade GSK-3β-phosphorylated KRAS protein. Knockdown of β-TrCP- and inhibition of GSK-3β abolish the combination treatment-induce KRAS ubiquitination and reduction in expression. Last but not least, combination treatment suppresses PI3K/Akt/m-TOR signaling pathway.

**Conclusions:**

Our data clearly show that the combination treatment significantly enhances KRAS protein degradation via the ubiquitination ubiquitin–proteasome pathway, which is also demonstrated in xenograft mouse model. The study provides strong scientific evidence for the development of the combination of WNT974 and ART as KRAS-targeting therapeutics for CRC treatment.

**Video Abstract**

**Supplementary Information:**

The online version contains supplementary material available at 10.1186/s12964-022-00834-2.

## Background

Colorectal cancer (CRC) is one of the most common malignant tumors of the gastrointestinal tract. It is a highly heterogeneous disease with diverse genetic background. KRAS (kirsten rat sarcoma viral oncogene) mutation is one of the dominant mutations in CRC, which accounts for 40% of all the CRC cases [1]. The most common KRAS mutations are at codon 12 and 13, followed by mutations at codons 61 and 146 [2].

KRAS is downstream of epidermal growth factor receptor (EGFR). Upon EGFR activation, the tyrosine kinase in the intracellular region phosphorylates and activates KRAS and hence activates the RAS-RAF-MAPK signaling pathway. The activated KRAS-GTP will then be hydrolyzed by GTPase and switches back to the inactivate KRAS-GDP state. Therefore, KRAS is switched between the active (KRAS-GTP) and inactive (KRAS-GDP) states. However, mutations in KRAS result in aberrant activation of the downstream RAS-RAF-MAPK or phosphoinositide 3-kinase pathways, regardless of the EGFR activation status [3, 4].

Since KRAS activity is regulated by farnesylation-mediated protein modifications [5], farnesyl transferase inhibitors have been developed. Although they were effective in preclinical models, they failed in the clinical studies [6, 7]. Therapeutic approaches also include targeting KRAS downstream signaling with kinase inhibitors for the rapidly accelerated fibrosarcoma, mitogen-activated protein kinase kinase and extracellular signal-regulated kinase. However, only a minority of these provides marginal survival advantages to the CRC patients carrying KRAS mutations; moreover, the treatments result in significant adverse events [8]. Furthermore, CRC patients harboring KRAS mutations are insensitive to the current anti-EGFR therapy [9], which further limits their treatment choices.

Up to present, no effective pharmacological inhibitors for the KRAS oncoproteins has been approved for cancer treatment, leading to the perception that KRAS proteins are 'undruggable'. Indeed, based on the structure and regulatory mechanisms of the KRAS, researchers have identified a number of challenges in targeting KRAS for disease treatments such as lack of binding sites on the KRAS protein surface for the binding of small molecule inhibitors [10–12]. Nevertheless, interrupting the KRAS-membrane interaction and disrupting the KRAS subcellular localization remain as attractive therapeutic strategies. Recent studies on the functionally relevant post-translational modifications of KRAS protein such as phosphorylation and ubiquitylation also suggest new opportunities to inhibit KRAS activity.

WNT974 (also known as LGK974) is a selective and orally bioavailable (PORCN) inhibitor. PORCN is a membrane-bound O-acyltransferase in the endoplasmic reticulum that adds the palmitoyl group to Wnt ligands, which is a necessary step for processing Wnt ligand secretion [13]. WNT974 inhibits PORCN and hence the secretion of Wnt ligands and interferes the Wnt-mediated signaling. WNT974 is developed to treat Wnt-driven cancers [14]. It shows significant effects in several cancer types including ovarian cancer [15], lung cancer [16], squamous cell carcinoma [17, 18], glioblastoma [19, 20] and colon cancer [21]. Indeed, WNT974 is in clinical trial phase I and phase II (NCT01351103, NCT02278133) for CRC treatment [22, 23].

Many bioactive compounds are identified from medicinal herbs [24]. *Artemisia annua* L. is a medicinal plant used to treat fever and chills. Artemisinin, is an bioactive compound isolated from *Artemisia annua* L. Artesunate (ART) is a water-soluble semi-synthetic derivative of artemisinin, which is approved for treating severe malaria cases [25]. ART is also known to specifically inhibits cancer cell growth but not the normal cells [26–29]. National Cancer Institute has also conducted a study and found that several cancer cells including CRC are sensitive to ART treatments. ART is now under clinical trials for treating patients with high-grade anal intraepithelial neoplasia and solid tumour [30].

In our study, we found that the combination of WNT974 and ART exhibited synergistic effect in reducing CRC growth. Interestingly, the combination treatment significantly enhanced KRAS protein degradation and suppressed PI3K/Akt/m-TOR signaling pathway that may underlie the synergistic effect of the combination treatment.

## Methods

### Reagents

Artesunate (ART) was provided from Kuming Pharmaceutical Co. Ltd. WNT974 was purchased from MedChemExpress company, Dulbecco's Modified Eagle's Medium, fetal bovine serum were purchased from Thermofisher Scientific Company. Antibodies against β-TrCP, GSK-3β, ANAPC2, GSK-3β, p-Akt (Ser473), t-Akt, p-PI3K (Tyr458), t-PI3K, p-mTOR (Ser2448), t-mTOR and β-Actin were purchased form Cell Signaling Technology (Danvers, MA). Antibodies against KRAS, NRAS, HRAS were purchased form abcam (USA). Mouse anti-rabbit IgG-HRP secondary antibody was purchased from San Cruz Biotechnology (Santa Cruz, United States of America).

### Cell lines and culture

HCT116, HT29, SW480, SW620, COLO325, COLO205, HCT15 and RKO cells were purchased from American Type Culture Collection (Manassas, USA). Cells were cultured in DMEM supplemented with 10% FBS in a humidified atmosphere containing 5% CO_2_ and 95% air at 37 °C. The medium was changed every three days, and cells were passaged using 0.05% trypsin/EDTA.

### Western blot analysis

Proteins were extracted from CRC cells with RIPA lysis buffer, followed by centrifugation at 13,500 rpm for 15 min at 4 °C. Protein concentration was measured using Pierce (R) BCA Protein Assay Kit, and equal amount of protein was separated on 10% SDS-PAGE and transferred to PVDF membranes. After blocking (5% skim milk powder in TBST, 20) for 1 h at room temperature, the membrane was then incubated with the respective primary antibody overnight at 4 °C. Afterward, the membrane was incubated with secondary antibody for 1 h at room temperature. All antibodies were diluted in TBS-Tween 20 containing 5% dry milk. The immune-reactive proteins were detected by enhanced chemiluminescence (ECL) using X-ray film and ECL reagent.

### KRAS activity

The ubiquitinated proteins were isolated using the UBIQAPTURE-Q® kit (UW8995, Enzo Life Sciences). KRAS protein activity was examined by the KRAS activation assay kit (ab211159, Abcam) following company’s instruction.

### Real time PCR analysis

RNA was extracted from CRC cells using TRIzol reagent (Invitrogen), and cDNAs were subsequently prepared by reverse transcription. RT Profiler PCR Array experiment was performed following the instruction of RT2 Profiler PCR Array kit (QIAGEN). Quantitative polymerase chain reaction (PCR) was performed using the Quantitect SYBR Green PCR Master Mix (Qiagen, Valencia, CA) with 1 μL cDNA in a final volume of 10 μL and the following primers at a final concentration of 1000 nM. Primers for ANAPC2 were 5′-GGCAGCAAGGACCTCTTCAT-3′ (forward) and 5′-CTTGCTCAGTTCCTCCAGGG-3′ (reverse). Primers for CUL7 were 5′-GTGGCATTGATACGCGCATT-3′ (forward) and 5′- CTCCAGTCGTGGCTTCTGTT -3′ (reverse). Primers for UBE2M were 5′-GCAGCAGAAGAAGGAGGAGG-3′ (forward) and 5′-GTAGGTGGAGCCGATGTAGC-3′ (reverse). Primers for RNF123 were 5′-ATCCAGGGTCACAGGCATTG-3′ (forward) and 5′-CCACGCCTTGCCATAATTCG-3′ (reverse). Primers for SYVN1 were 5′-CTGCCTCCTTTTCCTCCAGG-3′ (forward) and 5′-TCTGAGCTAGGGATGCTGGT-3′ (reverse). Primers for NEDD4 were 5′- AGCCAGAGTTCTGCAGGCCCT-3′ (forward) and 5′- GCTGGGAAGTCCGGCATGCA-3′ (reverse). Primers for β-Trcp were 5′- AAGCGAATTCTCACAGGCCA -3′ (forward) and 5′- TCCATCATAGGCCCCACTGA -3′ (reverse). Primers for PEAK were 5′- ACCAGTCTCGCCTTGCCCCA -3′ (forward) and 5′- GGGGAGCGGAATGGGATGCG -3′ (reverse). Primers for elF5A were 5′- CCTGGTGGGGGAGAAGGGGG-3′ (forward) and 5′-CCTGAGGAGGGGGCAGGTCC-3′ (reverse). Primers for RAK were 5′- TCCCAGCTCCATTTGATTTGTC-3′ (forward) and 5′- TGACCAGATCCCAATCGCTTC-3′ (reverse). Primers for SMURF-2 were 5′- GTGGTTGATGGATCTGGGCA-3′ (forward) and 5′- ACTGTCCACATGTTGCACCA-3′ (reverse). Primers for UBCH5 were 5′- AGCGCATATCAAGGTGGAGT-3′ (forward) and 5′- AGCTGAAGATGCAGATGTCCA -3′ (reverse). Amplification of the cDNAs was performed using the LightCycler 2000 instrument (Roche, Indianapolis, IN). The cycling conditions comprised a denaturation step for 15 min at 95 °C, followed by 40 cycles of denaturation (95 °C for 15 s), annealing (59 °C for 20 s), and extension (72 °C for 15 s). After amplification, a melting curve analysis was performed with denaturation at 95 °C for 5 s, then continuous fluorescence measurement was made from 70 to 95 °C at 0.1 °C/second. Each sample was amplified in duplicate.

### siRNA transfection

HCT116 cells or SW620 cells were seeded in 6-well plates. CUL7, ANAPC2, UBE2M, SYVN1, and RNF123 siRNA were diluted to 10 μM working concentration for transfection. Then, 5μL Lipofectamine® RNAiMax transfection reagent (Invitrogen) was added to 150μL medium without serum in one tube, and 3μL of prepared siRNA was added to 150μL medium without serum, and incubated for 20 min at room temperature before adding to the well and cultured for 24 h.

### Colony formation assay

siRNA ANAPC2 knockdown HCT116 and SW620 cells were seeded in 6-well plates at a density of 2 × 10^5^ cells per well in 2 mL medium. Cells were treated with ART, WNT974 and combination for 14 days until individual cells formed distinctly visible colonies. Then cells were stained with 50% methanol solution of 2% Methylene Blue. After washing, plates were air dried and digital images were taken.

### CRC-bearing xenograft mouse model

The six-week-old male BALB/c nude mice were housed in the Laboratory Animal House of Hong Kong Baptist University. The animal house is temperature-controlled with a 12 h light/dark cycle. Food and water were available ad libitum. Mice were adapted to the environment for one week before the study. The procedures of all in vivo studies have granted ethics approval by the Animal Experimentation Ethics Committee of the Hong Kong Baptist University. The mice were subcutaneously inoculated with 1 × 10^6^ HCT116 cells in the left armpit. Once tumors were palpable (~ 100 mm^3^), the tumor-bearing nude mice were randomly divided into groups with five mice in each group for further studies. (1) Vehicle group (daily i.p. saline), (2) ART group (daily i.p. 30 mg/kg of artesunate), (3) WNT974 group (daily i.p. 5 mg/kg of WNT974), (4) ART combined with WNT974 group (daily i.p. 30 mg/kg artesunate and 5 mg/kg WNT974), (5) 5-Fu group (daily i.p. 10 mg/kg of 5-Fu). The tumor size and body weight were monitored every day.

### Immunohistochemical staining

Paraffin sections of tumors were deparaffinized using xylene and then rehydrated by immersing in alcohol at series of concentrations. Endogenous peroxidase was quenched by 10 min incubation with 3% hydrogen peroxide. Blocking serum of 1:10 was used for blocking the non-specific bindings of epitopes. The sections were incubated with β-TrCP, GSK-3β and KRAS antibody of 1:100 at 4 °C overnight. Slides were then washed three times, incubated with a biotinylated secondary antibody (Santa Cruz) for 30 min, and then with peroxidase substrate for 10 min. The sections were finally washed, incubated in deionized water for 5 min, counterstained with hematoxylin before analyzing by microscopy (NIKON Eclipse ci, NIKON digital sight DS-FI2, Japan).

### Statistical analysis

The data were shown as mean ± standard errors with three independent experiments. Statistical analysis was carried out with GraphPad Prism 8.0 software. Data are taken as significance when p < 0.05.

## Results

### The combination of WNT974 and artesunate (ART) reduces KRAS protein level and activity in CRC cells

**Fig. 1:**
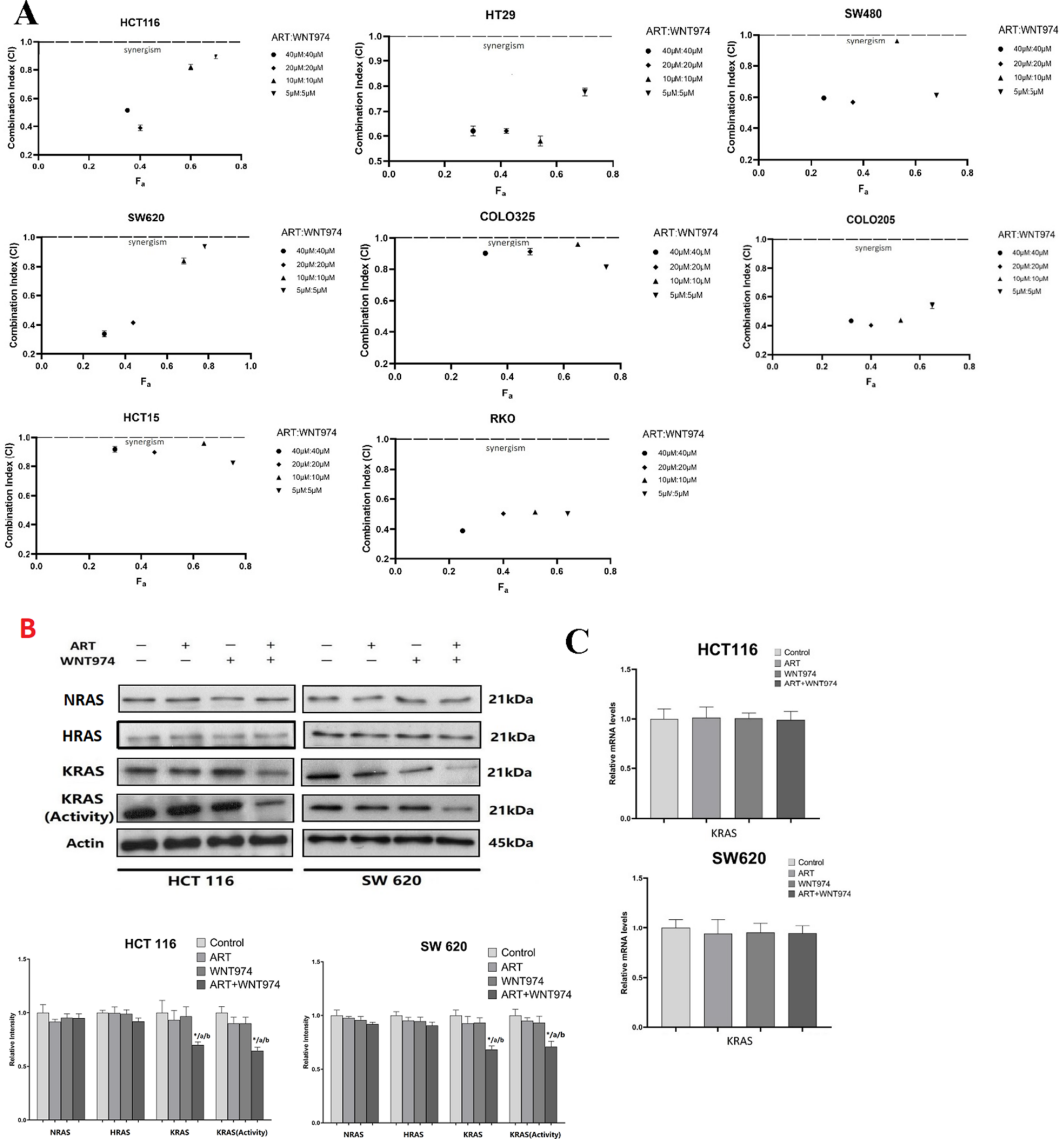
Combination of WNT974 and artesunate (ART) reduces KRAS protein level and activity in CRC cells. **A** Fraction affected (Fa) versus combination index (CI) plots were used to determine the extent of synergy for combination treatment in 8 CRC cells, synergistic effects are defined as CI < 1. **B** HCT116 and SW620 were treated with 20 μM WNT974, 20 μM ART or the combination of both for 48 h. The NRAS, HRAS KRAS protein level and KRAS activity were examined. **C** KRAS mRNA level in HCT116 cells and SW620 cells after treatments. Shown is mean ± SE, n = 3 individual experiments, *p < 0.05, compared to control; a < 0.05, compared to ART; b < 0.05, compared to WNT974

The combination of WNT974 and ART exhibited synergistic effect in reducing CRC cell viability (Fig. 1A) as indicated by the combination index (CI) < 1. Interestingly, the combination of WNT974 and ART significantly reduced KRAS protein levels (Fig. 1B) but did not affect NRAS and HRAS (Fig. 1B) in the CRC cells when compared to WNT974 or ART mono-treatments. Furthermore, the treatment did not affect the KRAS mRNA levels (Fig. 1C). We also examined the KRAS activity in these cells by using Raf1 RBD agarose beads to selectively pull-down the active form of KRAS from the protein samples. Figure 1B showed that the combination treatment significantly reduced KRAS activity in the CRC cells when compared to control or the monotreatments. These results imply that the reduced KRAS protein expression and activity may be associated with the synergistic effect of the combination treatment in reducing CRC growth.

### The combination of WNT974 and ART induces KRAS protein degradation in CRC cells

**Fig. 2:**
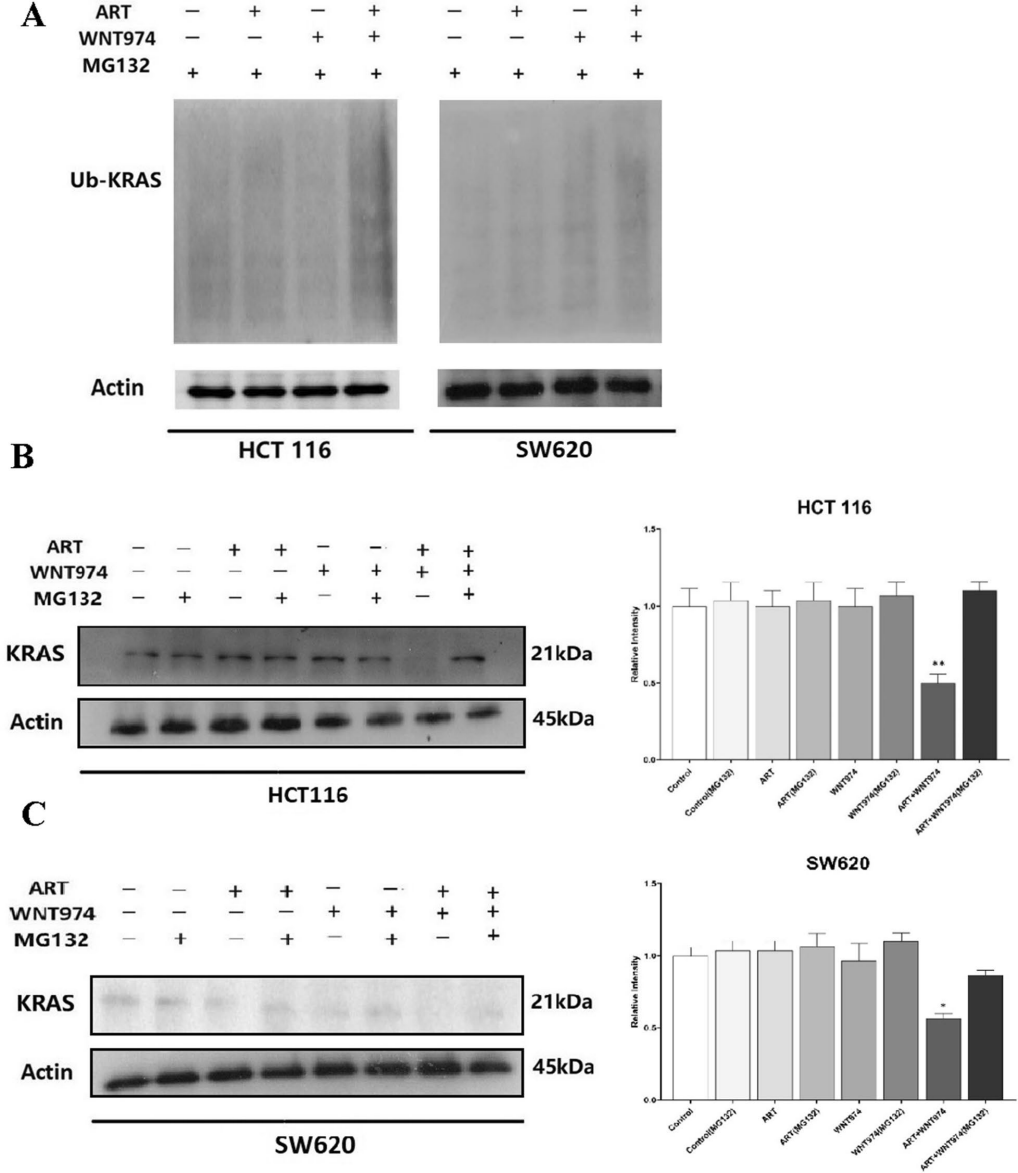
Combination of WNT974 and ART induces KRAS degradation. **A** HCT116 and SW620 were treated with 20 μM WNT974, 20 μM ART or the combination of both for 48 h. The ubiquitin-KRAS protein was examined by Western blot. The KRAS protein expressions in **B** HCT116 and **C** SW620 cells after the treatments in the presence or absence of MG132. Shown is mean ± SE, n = 3 individual experiments, *p < 0.05, **p < 0.01 compared to the combination treatment in the presence of MG132

Post-translational modifications of KRAS protein such as ubiquitylation and degradation may reduce the protein expression [31]. The ubiquitin proteasome pathway consists of concerted actions of enzymes that link chains of the polypeptide co-factor, ubiquitin, onto proteins to mark them for degradation [32]. We next examined whether the combination treatment affected KRAS ubiquitination. As shown in Fig. 2A, the combination treatment markedly enhanced KARS ubiquitination in the CRC cells when compared to the mono-treatments. To further examine whether the treatment affected KRAS protein degradation, we treated the cells with WNT974, ART or the combination of both in presence or absence of MG132. MG132 is a specific, potent, reversible cell-permeable proteasome inhibitor [33]. As shown in Fig. 2B and C, MG132 abolished the combination treatment-reduced KRAS protein expression, suggesting the combination of WNT974 and ART induces KRAS degradation via the ubiquitination proteasome pathway.

### The combination of WNT974 and ART induces KRAS protein degradation by increasing anaphase promoting complex subunit 2 (ANAPC2) expression

**Fig. 3:**
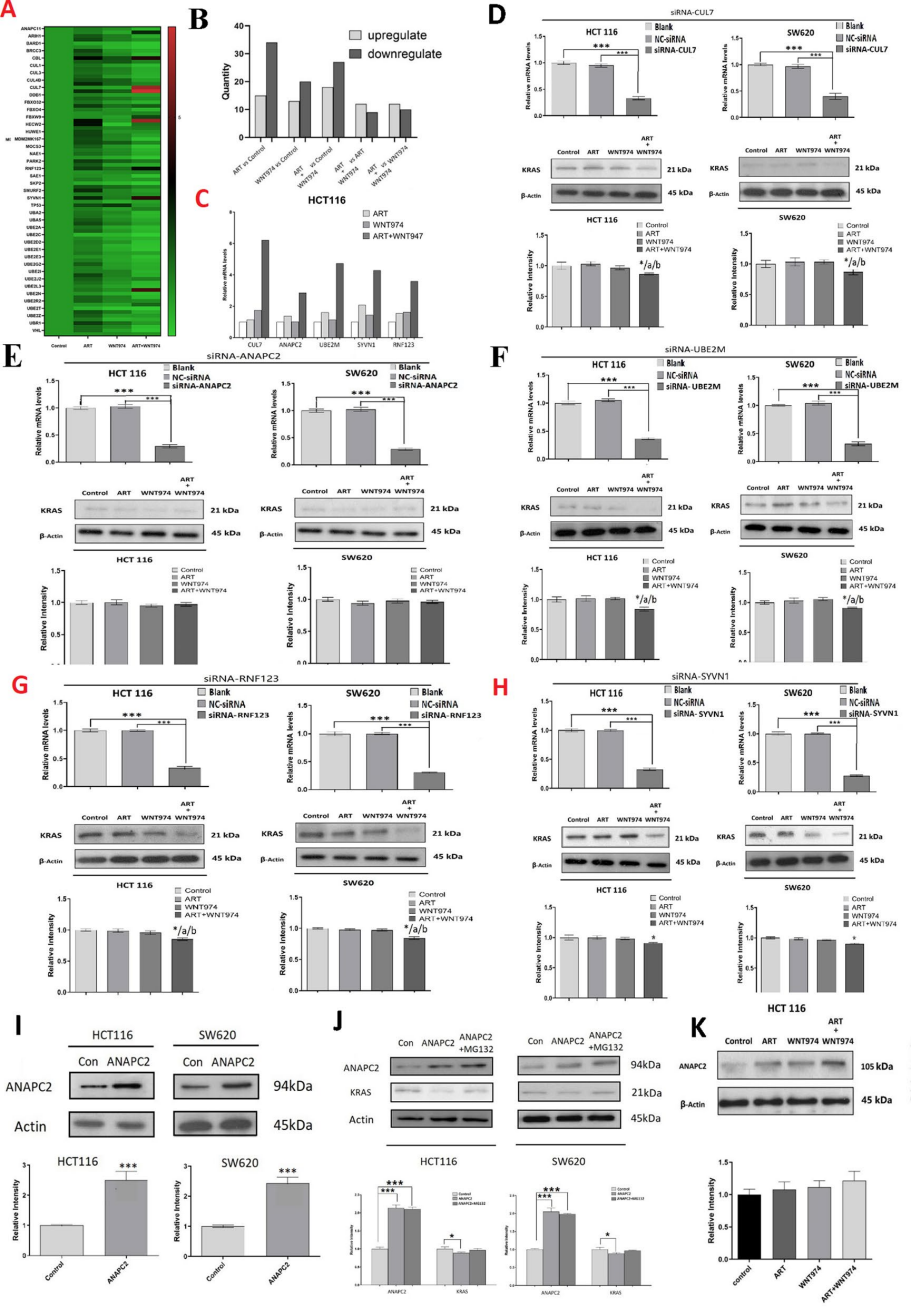
Combination of WNT974 and ART induces KRAS protein degradation by increasing anaphase promoting complex subunit 2 (ANAPC2) expression. **A** HCT116 cells were treated with 20 μM WNT974, 20 μM ART or the combination of both for 48 h. Heat map showing the PCR array results. **B** The numbers of up- or down-regulated genes in the PCR array. **C** The mRNA expressions of CUL7 (cullin), ANAPC2 (anaphase promoting complex subunit 2), UBE2M (ubiquitin conjugating enzyme E2M), SYVN1 (synoviolin 1) and RNF123 (RING finger protein123) in the CRC cells after the treatments. KRAS expression in the CRC cells after siRNA-mediated knockdown of **D** CUL7, **E** ANAPC2, **F** UBE2M, **G** RNF123, **H** SYVN1, upper panel showing the expressions of CUL7, ANAPC2, UBE2M, RNF123 and SYNV1 in these cells. **I** Protein expressions of ANAPC2 in ANAPC2-overexpressed CRC cells. **J** Protein expressions of KRAS in ANAPC2-overexpressed CRC cells in the presence or absence of MG132. **K** CRC cells were treated with 20 μM WNT974, 20 μM ART or the combination of both for 48 h. The protein expression of ANAPC2 after the treatments. Shown is mean ± SE, n = 3 individual experiments, *p < 0.05, *** < 0.001 compared to control; a < 0.05, compared to ART; b < 0.05, compared to WNT974

Next, we examined how the combination treatment induced KRAS degradation in CRC cells. Firstly, we used PCR array for the human ubiquitination pathway (genes layout in Table 1) to examine whether the treatments affected the expressions of the genes that are involved in the ubiquitination pathway. As shown in Fig. 3A, the heat map indicated that the treatments affected the gene expressions, the numbers of up- or down-regulated genes were shown in Fig. 3B. The results showed that, compared to control, ART treatment upregulated 15 genes and downregulated 34 genes; WNT974 treatment upregulated 13 genes and downregulated 20 genes; and the combination treatment upregulated 18 genes and downregulated 27 genes. Compared to ART treatment, the combination treatment upregulated 12 genes and downregulated 9 genes. Compared to WNT974 treatment, the combination treatment upregulated 12 genes and downregulated 10 genes. More importantly, we found that 5 genes were upregulated by more than twofold under the combination treatment when compared to the mono-treatments. These ubiquitination related genes were CUL7 (cullin), ANAPC2, UBE2M (ubiquitin conjugating enzyme E2M), SYVN1 (synoviolin 1) and RNF123 (RING finger protein123) (Fig. 3C),

**Table 1:** PCR array genes layout

	1	2	3	4	5	6	7	8	9	10	11	12
A	ANAPC11	ANAPC2	APIH1	ATG7	BARD1	BRCA1	BRCC3	BTRC	CBL	CDC34	CUL1	CUL2
B	CUL3	CUL4A	CUL4B	CUL5	CUL7	CUL9	DDB1	DZIP3	FBXO3	FBOX31	FBX04	FBXW10
C	FBXW9	HECW1	HECW2	HERC5	HUWE1	MARCH5	MDM2	MIB1	MOCS3	MUL1	NAE1	NEDD8
D	PARK2	RFWD2	RNF123	RNF148	SAE1	SKP1	SKP2	SMURF1	SMURF2	STUB1	SYN1	TMEM189
E	TP53	UBA1	UBA2	UBA3	UBA5	UBA6	UBE2A	UBE2B	UBE2C	UBE2D1	UBE2D2	UBE2D3
F	UBE2E1	UBE2E2	UBE2E3	UBE2G1	UBE2G2	UBE2H	UBE2I	UBE2J1	UBE2J2	UBE2K	UBE2L3	UBE2M
G	UBE2N	UBE2Q1	UBE2R2	UBE2S	UBE2T	UBE2W	UBE2Z	UBE4B	UBR1	UBR2	VHL	WWP1
H	ACTB	B2M	GAPDH	HPRT1	RPLPO	HGDC	RTC	RTC	RTC	PPC	PPC	PPC

To examine whether these gene candidates were involved in the combination treatment reduced KRAS protein expression, we used siRNA to mediate the knockdown of the gene candidates before the treatments. The upper panels in Figs. 3D–H showed the siRNA-mediated knockdown of the candidates (CUL7, ANAPC2, UBE2M, SYVN1, RNF123) in the CRC cells. We found that the combination treatment could significantly reduce KRAS protein level in the CUL7-, UBE2M-, SYVN1-, RNF123-knockdown cells (Fig. 3D, F–H). However, the combination treatment failed to reduce KRAS protein level in the ANAPC2-knockout cells (Fig. 3E), implying ANAPC2 was involved in the combination treatment-reduced KRAS protein expression.

Next we detect whether ANAPC2 would affect cell activities by combination treatment. Results showed combination treatment in ANAPC2-knockout cells group, the cell colonies and cell viabilities were significantly higher than those in normal cells group (Additional file 2: Fig. S2). It indicated that ANAPC2 plays an essential role in cell growth of combination treatment. Then We further validated the role of ANAPC2 in KRAS protein degradation. We found that ANAPC2 overexpression (Fig. 3I) significantly reduced KRAS protein levels (Fig. 3J), which was reversed in the presence of MG132 (Fig. 3J). Furthermore, we also found that the combination treatment significantly increased ANAPC2 protein expression when compared to the mono-treatments (Fig. 3K). Our data strongly suggest that the combination treatment increases ANAPC2 expression and hence increases KRAS protein degradation in the CRC cells.

### The combination of WNT974 and ART induces KRAS protein degradation by increasing β-TrCP and GSK-3β expressions

**Fig. 4:**
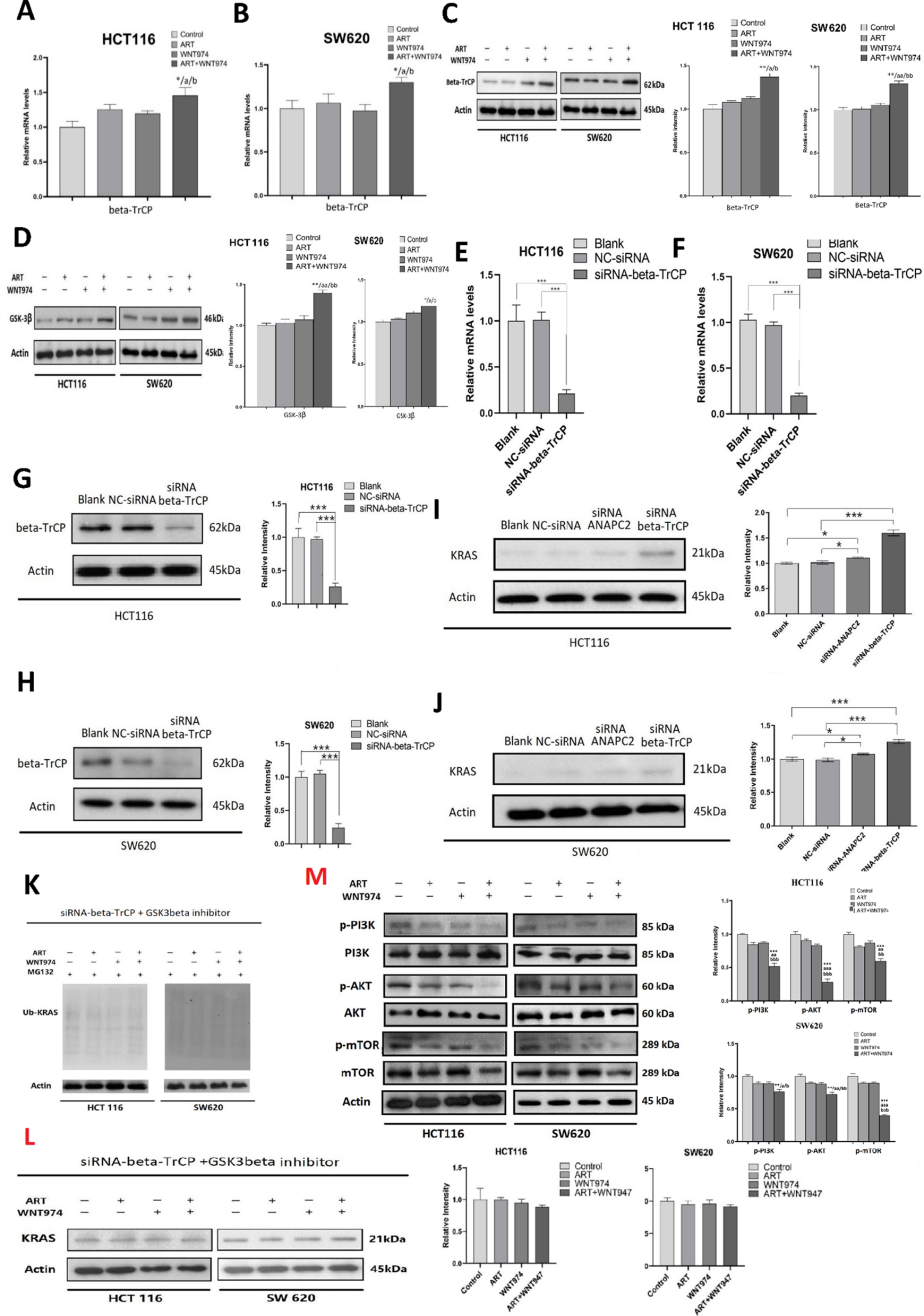
Combination of WNT974 and ART induces KRAS protein degradation by increasing β-TrCP and GSK-3β expressions. The mRNA level of β-TrCP in **A** HCT116 and **B** SW620 cells after treating with 20 μM ART, 20 μM WNT974 or a combination of both for 48 h. The protein level of **C** β-TrCP and **D** GSK-3β in the CRC cells after treating with 20 μM ART, 20 μM WNT974 or a combination of both for 48 h. The **E–F** mRNA expression and **G–H** protein expression of β-TrCP after siRNA-mediated knockdown of β-TrCP in the CRC cells. **I–J** Protein expression of KRAS in β-TrCP-knockdown or ANAPC2-knockdown cells. **K** Ubiquitination of KRAS in β-TrCP-knockdown cells after treating with 20 μM ART, 20 μM WNT974 or a combination of both treatments for 48 h, in the presence of GSK-3β inhibitor. **L** Protein expression of KRAS in the β-TrCP-knockdown CRC cells after treating with 20 μM ART, 20 μM WNT974 or a combination of both for 48 h, in the presence of GSK-3β inhibitor. **M** HCT116 and SW620 were treated with 20 μM WNT974, 20 μM ART or the combination of both for 48 h. The p-Akt (Ser473), t-Akt, p-PI3K (Tyr458), t-PI3K, p-mTOR (Ser2448), t-mTOR protein level were examined. Shown is mean ± SE, n = 3 individual experiments, *p < 0.05, **p < 0.01, *** *p* < 0.001 compared to control; a < 0.05, aa < 0.01, aaa < 0.001compared to ART; b < 0.05, bb < 0.01, bbb < 0.001 compared to WNT974

We further explored whether other gene candidates would affect KRAS expressions and degradation under the combination treatment. Other studies have reported that elF5A and PEAK increased KRAS protein synthesis; NEDD4 and β-TrCP which are E3 ligase will promote KRAS degradation; SMURF2 and UBCH5 as a critical E3:E2 complex maintaining KRAS protein stability [34–36]. In our study, we found that combination treatment did not significantly affect the mRNA expressions of elF5A, NEDD4, PEAK, SMURF2, UBCH5 (Additional file 1: Figure S1A to S1J), but significant increased β-TrCP mRNA level (Fig. 4A and B) and protein level (Fig. 4C) in the CRC cells. β-TrCP is an F-box ubiquitin ligase, it has been implicated in RAS (including KRAS) ubiquitination and degradation [35, 37, 38]. β-TrCP degrades KRAS protein and in this degradation process, glycogen synthase kinase-3 beta (GSK-3β) mediates the phosphorylation of KRAS for the priming of β-TrCP to the KRAS protein for degradation [35]. Interestingly, the combination treatment not only increased β-TrCP expression but also GSK-3β expression (Fig. 4D).

We then examined whether β-TrCP would affect the KRAS protein expression in the CRC cells.

We used siRNA to mediate the knockdown of β-TrCP in the CRC cells (Fig. 4E–H). We found that in the β-TrCP-knockdown cells, KRAS protein expression was increased (Fig. 4I and J). More importantly, in these cells, in the presence of GSK-3β inhibitor, the combination treatment failed to induce KRAS ubiquitination (Fig. 4K) and reduce KRAS protein expression (Fig. 4L). Taken together, the data strongly suggest that the combination treatment increases β-TrCP and GSK-3β expressions that lead to the KRAS degradation in CRC.

### The combination of WNT974 and ART suppressed PI3K/Akt/mTOR signaling pathway

PI3K/Akt/mTOR signaling pathway is a key downstream signaling pathway of KRAS. As we found combination treatment degraded KRAS protein, we further investigated whether combination treatment would affect PI3K/Akt/mTOR signaling pathway. As expected, our data showed that combination treatment reduced Akt, PI3K and mTOR phosphorylation in both the HCT116 and SW620 cells (Fig. 4M). These results suggest that the combination treatment suppressed PI3K/Akt/mTOR signaling pathway, which leads to the inhibition of tumor growth.

### The combination of WNT974 and ART exhibits a potent anti-CRC effect in vivo

**Fig. 5:**
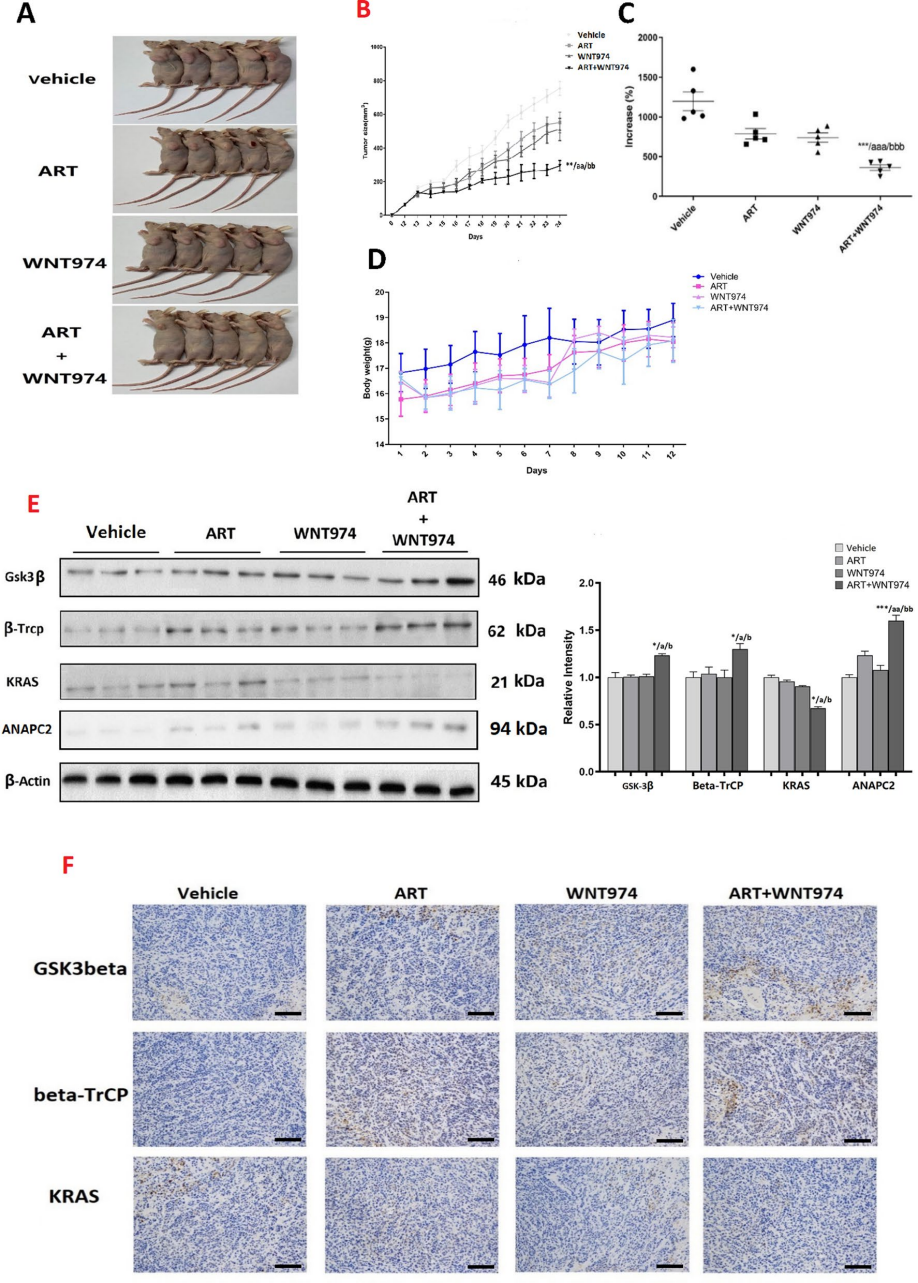
Combination of WNT974 and ART exhibits a potent anti-CRC effect in vivo. **A** The photographs of the CRC-bearing xenograft mouse model in different treatment groups. **B** Tumor volume, **C** % increase in tumor growth and **D** body weight of the mice. **E** Protein expressions of KRAS, β-TrCP, GSK-3β and ANAPC2 in the tumor tissues. **F** IHC analysis of the tumor tissues for KRAS, β-TrCP and GSK-3β (magnification × 200, Scale bars, 100 μm). Shown is mean ± SE, n = 5 mice in each group, *p < 0.05, **p < 0.01, *** *p* < 0.001 compared to control; a < 0.05, aa < 0.01, aaa < 0.001, compared to ART; b < 0.05, bb < 0.01, bbb < 0.001 compared to WNT974

Next, we examined the anti-CRC effect of the treatments with CRC-bearing xenograft mouse model. After 12-days of treatment, the combination treatment significantly reduces the tumor size (Fig. 5A and B) and the percentage increase in tumor size (Fig. 5C) when compared to the control and monotreatments. The combination treatment did not significantly affect the body weight of the mice (Fig. 5D), suggesting the treatments do not have apparent toxicity to the mice. In parallel with the in vitro data, the combination treatment significantly increased β-TrCP and GSK-3β expressions and reduced KRAS expression levels in the tumor tissues of these mice (Fig. 5E and F).

## Discussion

**Fig. 6:**
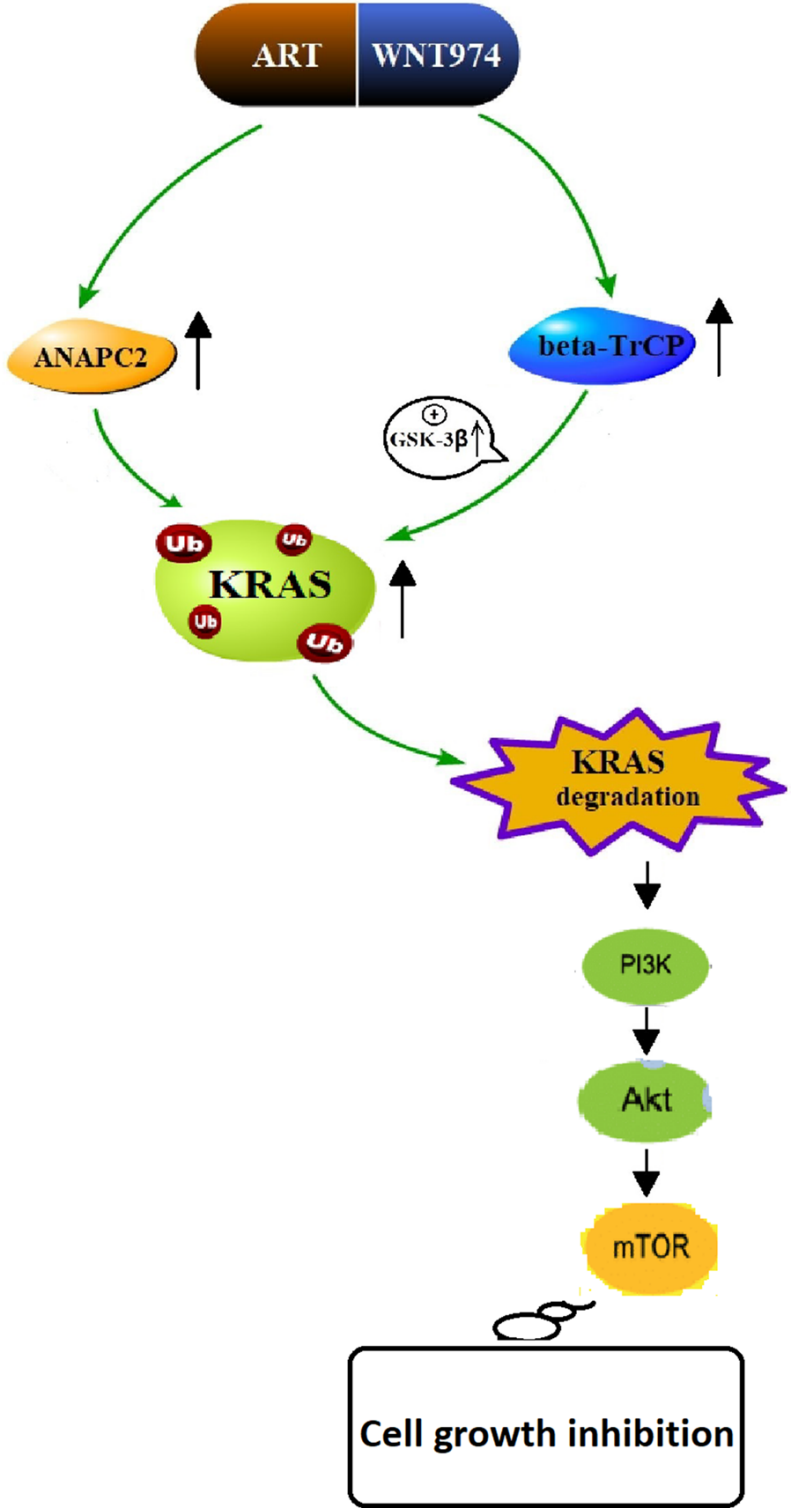
A schematic diagram showing the mechanism of action underlying the synergistic effect of the combination treatment. A schematic diagram showing the combination of WNT974 and ART increases ANAPC2, β-TrCP and GSK-3β expressions that lead to KRAS protein degradation via the ubiquitination ubiquitin–proteasome pathway. And then combination treatment inhibits CRC growth by suppressing PI3K/Akt/mTOR pathway

Our study shows that the combination of WNT974 and ART significantly inhibits CRC growth. Mechanistic studies suggest that the combination treatment reduces KRAS protein expressions by inducing KRAS protein degradation via the ubiquitination pathway, which may attribute to the elevated expressions of ANACP-2, beta-TrCP and GSK-3β. Then combination treatment suppresses PI3K/Akt/mTOR signaling pathway and lead to cell proliferation inhibition (Fig. 6).

The development of CRC is associated with the abnormal function of cellular transmembrane signaling systems [39]. KRAS is a well-known oncogene in CRC. It is susceptible to mutations. The most common KRAS gene mutation is at glycine at G12, G13 and glutamine at Q61, while G12 has 15 different point mutations. These mutation leads to the aberrant activation of the signaling pathways including the RAF/MEK/MAPK pathway, PI3K/Akt pathway and RAGDS, RAL-RLIP pathways, leading to the malignant CRC progression and development [40, 41]. Furthermore, KRAS mutation also influences the choice of surgical techniques and is an independent predictor for positive resection margins (HR 2.44, 95% CI 1.30–4.58, *P* = 0.005) [42].

Direct targeting KRAS mutant has been suggested as a therapeutic strategy. SCH-53239 was the first KRAS inhibitor that was designed to compete with GDP for the nucleotide binding site on the KRAS protein [43]. However, the efficacy was impeded by the fact that the hydrophobic pockets on the KRAS protein surface is not well-defined. Besides, the compound was toxic, and has not been approved by the Food and Drug Administration (FDA) for cancer treatment [44, 45]. Up to present, targeting KRAS for CRC treatment remains a clinical challenge [44]. Our data clearly show that the combination treatment significantly reduces CRC cell viability in eight different CRC cell lines and reduces KRAS expression and activity. These cells harbor different gene mutations including KRAS mutations in HCT116 and SW620 cells. Therefore, our data suggest that the combination treatment can be developed as KRAS-targeting therapeutics for treating CRC regardless of the mutation statuses.

Ubiquitination is an important protein posttranslational modification (PTM), it plays a crucial role in controlling protein degradation and maintaining a homeostasis [46]. Tumorigenesis involves many altered biological processes. Ubiquitination of some key signaling proteins such as RagA [47, 48], mTOR [49], PTEN [50, 51], Akt [52, 53], c-Myc [54, 55] and P53 [56, 57] have implicated in the tumor growth. Therefore, targeted ubiquitination and degradation of oncoproteins via the ubiquitin–proteasome pathway represents an alternative therapeutic strategy. By targeting the proteasome, E3 ligases, E1, E2 and deubiquitinases (DUBs), many targeted compounds have been developed to combat cancer, such as bortezomib, carfilzomib, oprozomib and ixazomib [58]. One of the most important components of the ubquitin conjugation machinery is E3 ligases that mediate protein degradation with high substrate specificity. Thus, targeting the active site of E3 enzymes or their interactions with substrates offers promising options for developing drugs with reduced side effects [59]. For example, Nedd4-1 is a general E3 ubiquitin ligase that controls the abundance of Ras. The interplay between Ras-regulated transcription of Nedd4-1 and Nedd4-1-mediated Ras degradation comprises a negative feedback regulatory loop [34]. Recently, it is found that KRAS is targeted for polyubiquitylation by E3 ligase β-TrCP and then subsequently degraded by the proteasomal degradation machinery [35, 37]. Our data show that the combination of WNT974 and ART increases β-TrCP expressions and hence KRAS protein degradation. Our study also, for the first time, revealing the regulatory role of E3 ligase ANAPC2 on KRAS protein degradation. ANAPC2 may be another therapeutic target that can increase KRAS protein degradation in CRC.

## Conclusions

In conclusion, we report a novel discovery for the combination of WNT974 and ART in inducing KRAS protein degradation in CRC. The combination treatment significantly increases the levels of E3 ligase ANAPC2 and β-TrCP and the expression of GSK-3β, which lead to KRAS protein degradation via the ubiquitination ubiquitin–proteasome pathway. And then we found combination treatment suppresses PI3K/Akt/mTOR signaling pathway which is KRAS downstream pathway. This study provides strong scientific evidence for the development of the combination of WNT974 and ART as KRAS-targeting therapeutics for CRC treatment.

## Supplementary Information

**Additional file 1**. Supplementary Figure 1.

**Additional file 2**. Supplementary Figure 2.

## Data Availability

The datasets used and/or analysed during the current study are available from the corresponding author on reasonable request.

## Abbreviations

CRC: Colorectal cancer
ART: Artesunate
MTT: 3-(4,5-Dimethylthiazol-2-yl)-2,5-diphenyltetrazolium bromide
RT-PCR: Real-time polymerase chain reaction
IHC: Immunohistochemistry
KRAS: Kirsten rat sarcoma viral oncogene
GDP: Guanosine diphosphate
GTP: Guanosine triphosphate
EGFR: Epidermal growth factor receptor
PORCN: Porcupine
DMEM: Dulbecco’s modified Eagle’s medium
FBS: Fetal bovine serum
PBS: Phosphate-buffered saline
cDNA: Complementary DNA
CUL7: Cullin
ANAPC2: Anaphase promoting complex subunit 2
UBE2M: Ubiquitin conjugating enzyme E2M
SYVN1: Synoviolin 1
RNF123: RING finger protein123
elF5A: Eukaryotic translation initiation factor 5A
NEDD4: Neuronal precursor cell-expressed developmentally down-regulated 4
PEAK: Pseudopodium enriched atypical kinase
SMURF2: SMAD Specific E3 Ubiquitin Protein Ligase 2
UBCH5: Ubiquitin Conjugating Enzyme E2 D1
