# Supplementary figures and images for: Combination of artesunate and WNT974 induces KRAS protein degradation by upregulating E3 ligase ANACP2 and β-TrCP in the ubiquitin–proteasome pathway
Source: Cell Commun Signal. 2022 Mar 19;20:34. doi: 10.1186/s12964-022-00834-2 (PMC8934478; doi:10.1186/s12964-022-00834-2)

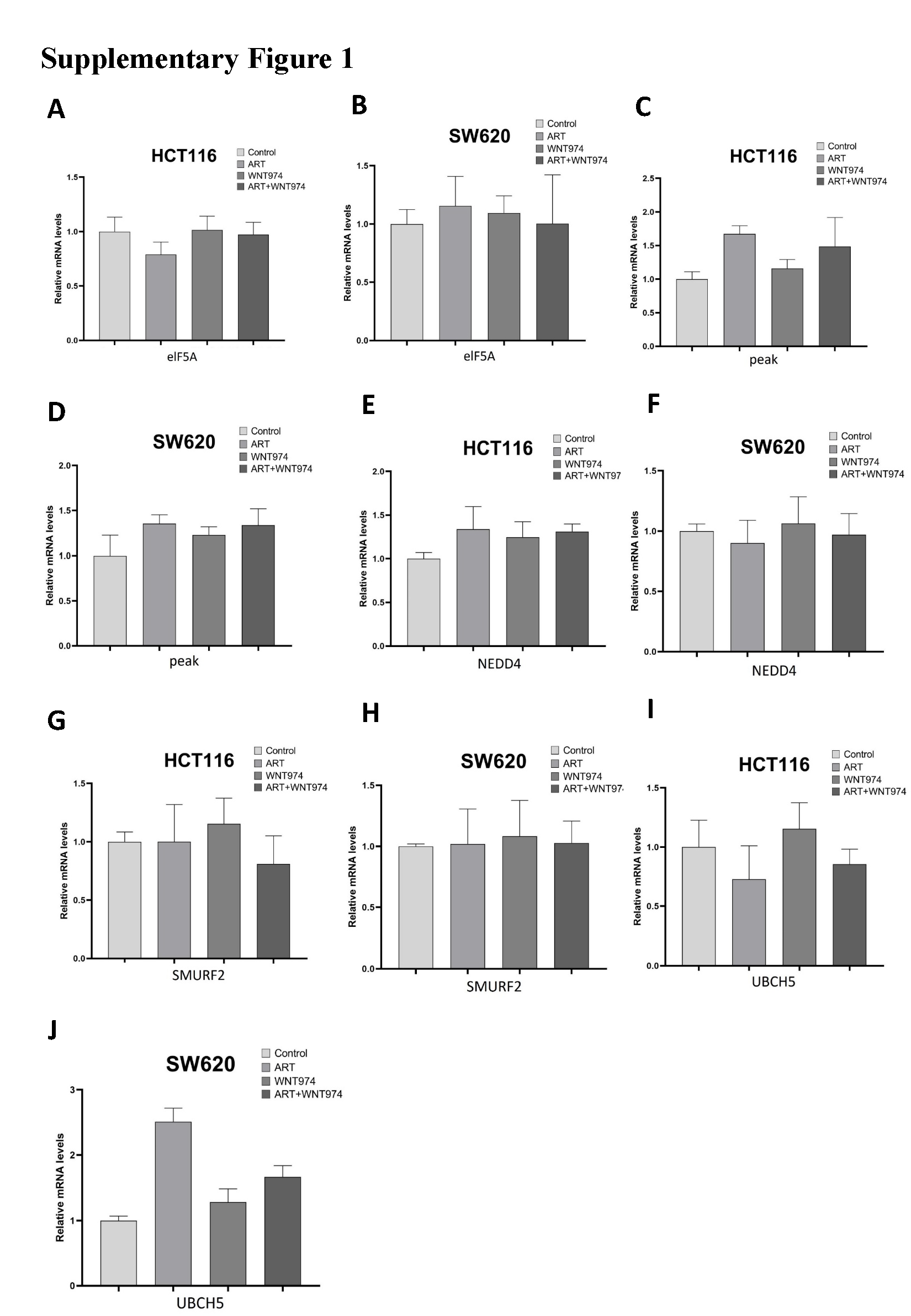

Supplement: Supplementary file 2 — Additional file 1. Supplementary Figure 1. [file 12964_2022_834_MOESM2_ESM.tiff]

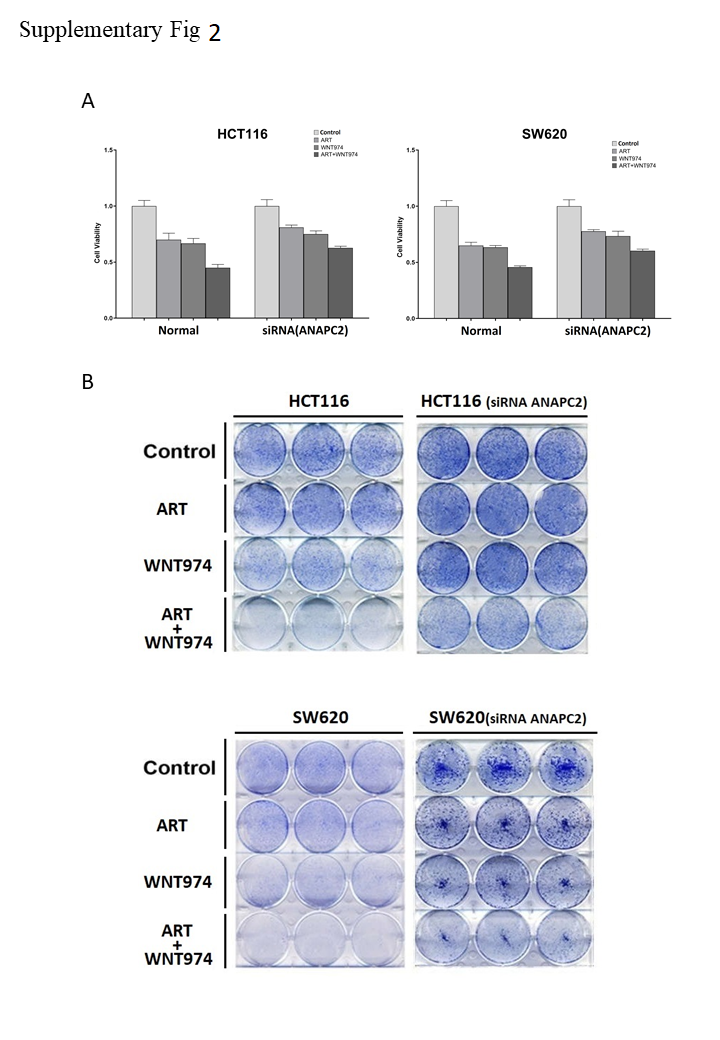

Supplement: Supplementary file 3 — Additional file 2. Supplementary Figure 2. [file 12964_2022_834_MOESM3_ESM.tif]
